# Heart on the left, diaphragm on the right: A case of congenital diaphragmatic eventration

**DOI:** 10.1002/ccr3.5068

**Published:** 2021-11-22

**Authors:** Sidra Naz, Vikash Jaiswal, Amey Joshi, Furqan Ahmad Jarullah, Esha Jain, Asmita Neupane

**Affiliations:** ^1^ University of Health Science Lahore Pakistan; ^2^ Larkin Community Hospital South Miami Florida USA; ^3^ Manipal Hospital Bangalore Karnataka India; ^4^ Jinnah Medical and Dental College Karachi Pakistan; ^5^ Kathmandu Medical College Teaching Hospital Kathmandu Nepal

**Keywords:** congenital abnormalities, diaphragmatic eventration, malformation, pediatric infection

## Abstract

Congenital Diaphragmatic Eventration (DE) requires a prompt diagnosis to avert the potentially life‐threatening complications. Herein, a 5‐month‐old male presented with recurrent respiratory infections due to a right‐sided diaphragmatic eventration. Misdiagnosed from previous medical visits, timely surgical intervention by thoracoscopic plication of the diaphragm was crucial for our patient's survival.

## Graphical abstract

Diaphragmatic eventration (DE) is a life‐threatening abnormality that leads to one or both hemi‐diaphragms being elevated in the thoracic cavity. Congenital DE can fatally hinder lung formation. DE can be easily misdiagnosed, thus recognizing the presentation, and performing accurate interventions can allow for suitable management and favorable outcomes.

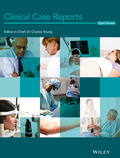

## 1 INTRODUCTION

Diaphragmatic eventration (DE) is a rare disease entity defined as a partial or complete elevation of one or both the hemidiaphragm, due to muscular or nervous dysfunction.^1^ DE can present as a congenital defect due to paucity or absence of varying degrees of muscle fibers or, acquired due to phrenic nerve injury.^2^ This report highlights the case of a right‐sided DE in a 5‐month‐old male infant presenting with recurrent lower respiratory tract infections and ongoing pneumonia.

## 2 CASE REPORT

A 5‐month‐old male infant presented to the University of Health Science, Pakistan, with recurrent episodes of fever and non‐productive cough which had increased in frequency in the last month. The infant was full‐term and delivered via spontaneous vaginal delivery at home to a 30‐year‐old mother. The mother was screened and found negative for HIV and hepatitis panel and denied any use of alcohol, tobacco, and illicit substances throughout the course of her pregnancy. The infant weighed 2500 g at birth; cried immediately after birth and has been breastfeeding without signs of distress. The infant had been meeting the required motor and language milestones along with good social maturation. Family history was significant for a consanguineous marriage along with the uneventful spontaneous vaginal delivery of two male babies who are currently healthy. The patient was immunized as per the national immunization schedule.

**FIGURE 1:**
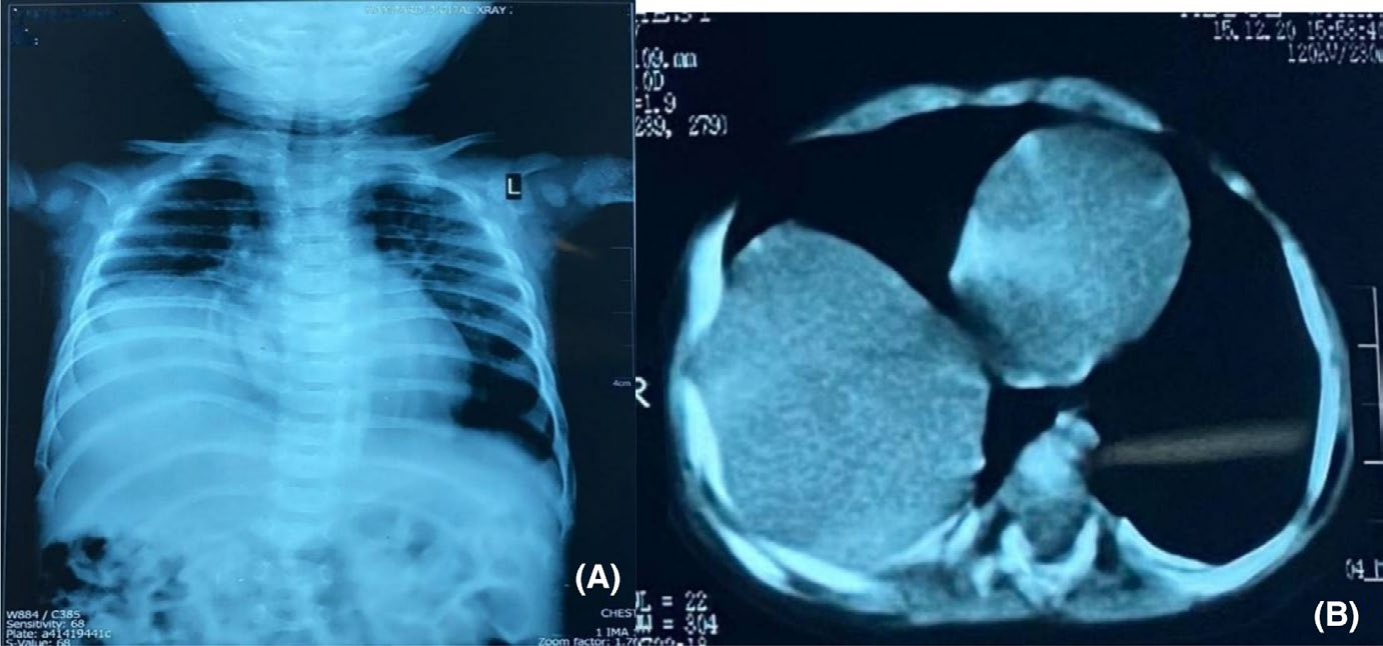
(A) CXR shows eventration on the right hand side revealing and elevated dome of the diaphragm. (B) CT scan showing liver on the right side adjacent to the heart in a higher position than normal

On admission, the infant was found to be irritable and inconsolable. General physical examination was significant for a fever (102°F). Chest examination revealed decreased movements and breath sounds in the right infra‐mammary, infra‐axillary, and infra‐scapular areas. Upon auscultation, crepitations were present in the right lung field. Initial laboratory investigations displayed an elevated total leukocyte count of 15,000/mm^3^. Inflammatory markers such as C‐reactive protein (CRP) and erythrocyte sedimentation rate (ESR) were negative. Hemoglobin and metabolic panel were also within normal limits. Chest X‐ray (CXR) was performed and showed that the dome of the diaphragm was raised on the right hand side as seen in Figure 1A. CT scan of the chest confirmed the diagnosis of a right‐sided diaphragmatic eventration with consolidative changes visualized in Figure 1B.

To treat ongoing pneumonia, the infant was empirically started on injection ampicillin, cloxacillin, and cefotaxime. He was also treated with albuterol and ipratropium bromide nebulization, dexamethasone, antipyretics, and multivitamin supplementation. Thoracoscopic plication of the diaphragm of the right side was planned. Under general anesthesia, the infant was placed on the left lateral decubitus position and a 3mm port was inserted in the 4th intercostal space at the posterior axillary line. A second 3mm incision in the 6th intercostal space of the right anterior axillary line was used to insert the Maryland grasper. Upon visualization of the diaphragm, right‐sided eventration was confirmed. There were no adhesions with abdominal viscera present. The diaphragm was plicated using 2–0 silk. Chest tube of appropriate size was placed in the right 5th intercostal space and closure was done in layers.

The immediate postoperative period was uneventful and the infant was able to maintain normal oxygen saturation on room air. Repeat CXR displayed a flattened hemidiaphragm on the right side. After successful management the infant was discharged and was symptom‐free at his 3‐month follow‐up appointment. CXR was repeated and was unremarkable showing adequate inflation of bilateral lungs and flattened diaphragm.

## 3 DISCUSSION

Diaphragmatic eventration, often described as the neurogenic muscular aplasia of the diaphragm, has been found to occur in varying age groups of the population.^1^ Congenital diaphragmatic eventration can be attributed to the inability of myoblasts to properly migrate to the septum transversum, thus leading to the substitution of muscle fibers by fibroelastic fibers.^3^ This histopathological picture of the diaphragm can be used to differentiate it from acquired causes of DE including birth trauma, phrenic nerve injury, nerve compression, pneumonia, and multiple sclerosis.^1^, ^4^

On top of the challenging presentation of DE, barriers to health care such as poor socioeconomic status, add to the complications. Home‐deliveries carried out by midwives have become a cultural norm in Pakistan in both rural and urban settings and have been correlated with a higher risk of maternal mortality.^5^ These practices have been attributed to the low socioeconomic status and deep‐rooted cultural beliefs prevailing in these regions. The delayed diagnosis of DE in the present case, similarly, may be due to the family's psychosocial views and inaccessibility of the medical healthcare. Recognizing cultural limitations is necessary as DE has widely been underreported due to its largely asymptomatic presentation. A summary table of our patient Physical exam findings has been presented in Table 1.

**TABLE 1:** Examination findings on admission/Pre‐op

Examination findings seen generally in a case of DE	Examination findings observed in the reported case of DE
±Fever Tachypnea (>60/min) ±Central cyanosis (seen in bilateral DE) ±Hypoxemia Failure to thrive ±Accessory muscle use ±Subcostal retractions ±Paradoxical chest movement Decreased tactile fremitus on affected side Decreased breath sounds on affected side Dullness to percussion on affected side Wheezing ±Tracheal shift due to mediastinal shift ±Scaphoid abdomen	Febrile (102°F) Respiratory rate of 40/min No cyanosis Hypoxemia present (SpO_2_: 88% at room air) No failure to thrive No accessory muscle use Subcostal retractions present No paradoxical chest movement Decreased tactile fremitus on affected side Decreased breath sounds on affected side Dullness to percussion on affected side Wheezing and crepitations present on the affected side. No tracheal shift Shape of abdomen was flat with no signs of trauma.

The non‐contracting, higher position of the diaphragm results in lung collapse followed by atelectasis predisposing the individual to bronchial or parenchymal infections. Presenting symptoms often include cough, dyspnea, chest pain, and cyanosis, and at times, gastrointestinal symptoms such as nausea, vomiting, abdominal pain, and acid reflux. In infants, these symptoms can be extremely debilitating due to the underdeveloped thoracic cage and intercostal muscle weakness resulting in paradoxical respiration ultimately necessitating the use of mechanical ventilation.^6^

The workup of DE primarily includes imaging modalities including the use of ultrasonography, chest x‐ray, and computed tomography (CT) scan. It is vital to exclude intrathoracic, mediastinal, or abdominal masses such as diaphragmatic hernia before concluding the diagnosis of DE.^7^ Fluoroscopic sniff test has been used to differentiate diaphragmatic eventration and paralysis in cases of unilateral diaphragmatic paralysis, however, this test was not used in our case.^1^ As recurrent lower respiratory tract infections are a common complexity arising from DE, it is of primary interest to treat the underlying infection and provide respiratory support with oxygen supplementation in patients presenting with hypoxia. A summary of previously published cases has been presented in Table 2. Nutritional supplementation is vital in infants presenting with DE as undernourishment due to poor feeding often accompanies the condition. Symptomatic DE presenting with respiratory distress, failure to thrive, recurrent pneumonia, and failure to wean of ventilator support have been shown to benefit greatly with diaphragmatic plication with a good prognosis and improvement of quality of life.^1^, ^8^

Diaphragmatic plication has been achieved through thoracotomy, thoracoscopic, and laparoscopic surgeries. Thoracoscopic surgeries have been proven to be more advantageous with a lower length of hospital stay, lower rate of complications, and better prognosis.^2^ Laparoscopic surgeries are also associated with lesser pain as intercostal nerve damage is avoided. The placement of a prosthetic mesh has been successful along with plication in cases of extreme amyotrophy, however at the cost of increased cost and chance of infection.^9^ Possible complications of diaphragmatic plication include pneumonia, dyspnea, pulmonary edema, and pleural effusion.^6^ These complications, however, were not observed in our case.

**TABLE 2:** Summary of published cases with common surgical procedures and outcomes in patients with Diaphragmatic Eventration

First author et al	Year of publication	Country	Age	Gender	Comorbid	Symptoms	Diagnostic criteria	Final Diagnosis	Surgical Management	Medical Management	Sepsis	Outcome (Dead/Survived)
Mouroux J. et al.^10^	2005	France	Mean age = 57.7 ± 14.8 years	4 males & 8 females	Trauma (n = 9), Charcot‐ Marie disease (n = 1), calcified para‐aortic nodes (n = 1)	Dyspnea (n = 12), palpitations (n = 4), chest pain (n = 3), dyspepsia (n = 2) & recurrent pneumonia (n = 1)	CT upper abdomen, MRI and/or phrenic electromyography	Diaphragmatic Eventration	VATS with 2 thoracoports & 4cm mini‐ thoracotomy‐Diaphragmatic plication	Post‐op follow‐up included physical exams, chest roentgenogram & spirometry at 3, 6 & 12 months	None	Survived (n = 12)
Shwaartz C., et al.^7^	2017	USA	31	male	Hypertension	Palpitations, shortness of breath & chest pain	Chest Radiograph, CT Abdomen, Barium enema	Left Diaphragmatic Eventration	Laparoscopic exploration and mesh removal with left thoracotomy, diaphragmatic plication	Post op radiograph, Follow‐up at 1 and 3 weeks	None	Survived (n = 1)
Zhao S., et al.^11^	2020	China	Median age: 12.2 months	90 male, 35 female	19 with congenital heart disease, 16 with congenital pulmonary dysplasia, 8 with pectus excavatum, 4 with hiatal hernia, 3 with pectoral malformations	Cough, asthma, dyspnea, recurrent respiratory tract infections, milk refusal, vomiting & arrhythmia	CXR, CT or GI radiography	Congenital Diaphragmatic Eventration	Thoracotomy on R. diaphragmatic eventration & laparotomy for Left Diaphragmatic Eventration. Transthoracic diaphragm plication & transabdominal diaphragm plication	Yearly radiological exams	None	Survived (n = 124) Died (n =1)
Omenai SA., et al.^12^	2020	Nigeria	69	male	Intestinal malrotation, renal agenesis, thoracoabdominal compartment syndrome, dilated cardiomyopathy	Easy fatigability, orthopnea, paroxysmal nocturnal dyspnea, pedal swelling, worsening breathlessness, early satiety and abdominal pain	ECHO, Autopsy	Thoracoabdominal compartment syndrome due to right hemidiaphragm eventration	N/A	N/A	N/A	Died
Shaher A et al.^13^	2019	Saudi Arabia	Early 20	male	No known comorbids	Shortness of breath, abdominal distention	Chest radiograph, acidic pH, lactate 8 mg/dl	Right‐ sided Diaphragmatic Eventration	The patient was shifted to OR, norepinephrine infusion was started. Midline laparotomy was done and a huge colon was encountered and eviscerated	Patient was resuscitated with 4L of 0.9% normal saline with no urine output, and intubated with minimal dose sedation (25 mcg fentanyl)	Septic Shock	Died
Wu S. et al.^14^	2015	China	10.28 ± 2.35 months	128 male, 49 female	Hypoplastic lung, congenital heart disease, cryptorchidism	asymptomatic, Rapid breathing, vomiting, recurrent respiratory infections	CT Abdomen, ECHO, CXR	Congenital Diaphragmatic Eventration	Diaphragmatic Plication	No recurrence at annual follow‐up	None	Survived (n = 177)
Carrasco A. et al.^6^	2018	Peru	17	female	Thoracic renal ectopia	Dry cough, chest pain, respiratory distress, bronchial spasms, repetitive episodes of bronchial asthma	CT, CXR	Diaphragmatic Eventration	Laparoscopy, posterolateral thoracotomy, hemidiaphragm plication		None	Survived
Kang H., et al.^15^	2019	Korea	28 months	male	osteochondroma, premature,	Asymptomatic	CXR, Fluoroscopy and ultrasonography	Congenital diaphragmatic eventration		N/A	None	Survived
Boufidou A., et al.^16^	2011	Greece	70 years old	female	N/A	Retrosternal, stabbing pain with radiation to precordial area	CXR, thoracic CT	Diaphragmatic eventration	?	No recurrence of symptoms	none	Survived
Guzman JPS., et al.^17^	2017	Philippines	32	female	None	Intermittent dyspnea, epigastric discomfort	CXR, CT chest	Congenital left diaphragmatic eventration	Diaphragmatic eventration via abdominal approach	Incentive spirometry, deep breathing exercises, 2 year follow‐up no symptoms	None	Survived
Deveer M., et al.^18^	2013	Turkey	64	male	None	Sudden onset severe dyspnea after strong cough	CT Thorax	Diaphragmatic eventration	Laparoscopy	N/A	None	Survived
Gunadi., et al.^19^	2020	Indonesia	16 days old	male	none	Respiratory distress, decreased breath sounds	CXR, CT	Congenital diaphragmatic eventration	Hemidiaphragm plication	Mild cough at 6 month follow‐up	None	Survived
Chowdhury S., et al.^20^	2018	Saudi Arabia	48 yo	Male	None	Spontaneous breathing, decreased air entry, increased respiratory rate	CXR, FAST, chest CT	Diaphragmatic eventration	Patient refused	No complaints on 11th day post admission	None	Survived
Kasdallah N., et al.^21^	2017	Tunis	4 days old	male	Neonatal gastric perforation	Bilious vomiting, refusing feeds, jaundice, respiratory distress	CXR, ultrasound	Congenital diaphragmatic eventration	Laparotomy & diaphragmatic plication	Infant well at 15 months old	None	Survived
Joshi A et al.^22^	2018	India	4 days old	female	None	Breathing difficulty since birth	CXR, ECHO	Left congenital diaphragmatic eventration. Followed by right‐sided eventration	Laparotomy & diaphragmatic plication, followed by right thoracotomy	(Synchronized Intermittent Positive Pressure Ventilation, followed by CPAP and PEEP)	After recurrence but on left side with GBS	Survived
Rajkumar JS., et al.^23^	2017	India	28 yo	female	30 weeks gestation,	Acute respiratory distress, decreased breath sounds	MRI	Diaphragmatic eventration	4 port technique with thoracoscopic diaphragmatic plication	Mom and baby well 2 months after surgery	None	Survived
Makwana K., et al.^24^	2017	India	58	female	None	Fever, cough, yellowish expectoration for 1 week	CXR, CT chest, PET/CT,	Diaphragmatic Eventration	Diaphragmatic plication	n/a	none	Survived
Pradhan P., et al.^25^	2020	Nepal	47	female	Typhoid fever at 17	1 year of abdominal pain, bloating & fullness after meals	CXR, CT chest/abdomen, chest ultrasound	Left hemidiaphragm eventration	Left hemidiaphragm plication via mini thoracotomy of left thorax	No symptoms at 1 month follow‐up	none	Survived
Dontukurthy S., et al.^26^	2020	USA	46	female		Shortness of breath, food intolerance, inability to sleep supine for 1.5 years	Chest radiograph, CT scan	Congenital diaphragmatic eventration	Diaphragmatic plication		None	Survived
Rajkumar JS., et al.^23^	2017	India	28	female	30 weeks pregnancy	Acute onset respiratory distress	MRI chest	Huge eventration of the right dome of diaphragm	Thoracoscopic diaphragmatic plication	None	None	Survived
Al‐Zayer F., et al.^27^	2019	Saudi Arabia	27	female	NKCM	Respiratory distress post elective cesarean section	ECG, CXR, Abdominal CT	Right diaphragmatic herniation	Right posterolateral thoracotomy	None	None	Survived
Pradhan P., et al.^28^	2020	Nepal	47	Female	Bilateral foot drop since 30 years	Abdominal distension, pain and bloating after meals	CXR, CT abdomen	Eventration of left hemidiaphragm	Left mini thoracotomy	None	None	Survived
Vinod Kumar MS., et al.^29^	2018	India	5	Male	NKCM	Abdominal pain, vomiting, constipation, fever	CXR, CT chest	Left sided diaphragmatic hernia	Laprotomy with left subcoastal incision	None	None	Survived
Stamenovic D., et al.^30^	2017	Germany	60	female	Right leg amputation secondary to arterial embolism	Chronic assisted ventilation	‐	Diaphragmatic eventration	Double‐lined diaphragmatic plication by means of uniportal video‐assisted thoracic surgery technique	None	None	Survived
Li XS., et al.^31^	2021	China	24	male	Neurofbromatosis type 1	Spontaneous pain and swelling of left upper abdomen	CXR, CT chest, biopsy of diaphragm	a diaphragmatic hernia caused by spontaneous diaphragmatic rupture	Diaphragmatic folding	None	Just fever	Survived
Manson HJ., et al.^32^	2017	UK	30	female	Dyspepsia secondary to gastric herniation	Worsening abdominal pain	CT chest, abdomen and pelvis	Congenital diaphragmatic hernia	Needle thoracostomy, laprotomy, total gastrectomy with Roux‐en‐Y reconstruction and splenectomy, sutured repair of the defect in the left hemidiaphragm	Analgesia, antiemetics	None	Survived
Fujii T., et al.^33^	2019	Japan	72	male	Gastric cancer of antrum	Abdominal pain	CXR, upper GI endoscopy	Left sided diaphragmatic eventration	Laparoscopic distal gastrectomy followed by diaghramatic plication	None	None	Survived
Glasberg T., et al.^34^	2017	USA	1 day	female	Preteerm baby, Polyhydroamnios 1 week prior to delivery	Respiratory insufficiency	CXR, ECHO, abdominal US	Pulmonary hypoplasia, hepatomegaly	‐	Multiple pressors, fluid resuscitation, optimizing ventilator	None	Died
Sharan KV., et al.^35^	2021	India	47	male	Breathlessness, left sided chest pain and fever	None	CXR, CT thorax	Hepatodiaphragmatic interpostition	Thoracoscopic diaphragmatic plication	Antibiotics and nebulization	None	Survived
DiChiacchio L., et al.^36^	2018	USA	3 days	male	Recurrent, chest infections	Preterm, recurrent pneumonias	CXR, CT angiogram chest	Extrapulmonary versus intrapulmonary sequestration with a systemic feeding vessel from the left internal mammary artery	Video assisted thoracoscopic resection	o	None	Survived

Symptomatic diaphragmatic eventration is associated with high morbidity and failure to thrive if not treated promptly. Adequate workup to exclude underlying conditions, nutritional status, associated abnormalities, and treatment of the same is vital to ensure better survival rates in infancy. Minimally invasive procedures like laparoscopic plication of the diaphragm are found to be very effective in the treatment of diaphragmatic eventration with a low incidence of complications and a good prognosis.

## 4 CONCLUSION

Congenital diaphragmatic eventration (DE) is a rare pathology that can be fatal if left untreated. DE’s are difficult to diagnose as they can present without symptoms thus requiring intricate management. Infants dealing with DE are at an increased risk of morbidity as their thoracic cage is underdeveloped leading to life‐threatening complications including failure to thrive. This case demonstrates the successful outcome of the patient due to accurate diagnosing of congenital DE, and the performance of minimally invasive procedures such as laparoscopic plication.

## CONFLICT OF INTEREST

None declared.

## AUTHOR CONTRIBUTIONS

SN, VJ, AJ, FAJ: wrote the initial draft of the manuscript; AN, ES: reviewed the manuscript. VJ, ES, FAJ: edited the draft and reshaped it into this manuscript; all authors approved the final version of the manuscript and agree to be accountable for all aspect of the work in ensuring that question related to the accuracy or integrity of any part of the work are appropriately investigated and resolved.

## ETHICAL APPROVAL

Written informed consent was obtained from the patient for publication of this report and any images related to the patient. A copy of the consent is available for review by the Editor in Chief of the journal.

## CONSENT

Written informed consent was obtained from the patient’s guardians for publication of this case report and any accompanying images. A copy of the written consent is available for review by the Editor‐in‐Chief of this journal.

## Data Availability

Data sharing is not applicable to this article as no new data were created or analyzed in this study.
